# Improved antimelanogenesis and antioxidant effects of polysaccharide from *Cuscuta chinensis* Lam seeds after enzymatic hydrolysis

**DOI:** 10.1590/1414-431X20187256

**Published:** 2018-05-28

**Authors:** Zi-Jun Liu, Ya-Lan Wang, Qi-Ling Li, Liu Yang

**Affiliations:** School of Traditional Chinese Medicine, Southern Medical University, Guangzhou, China

**Keywords:** *Cuscuta chinensis* polysaccharide, Antimelanogenic activity, Enzymatic hydrolysis polysaccharide, Antioxidant activity

## Abstract

*Cuscuta chinensis* polysaccharide (CPS) was extracted using hot water and enzymatically hydrolyzed *C. chinensis* polysaccharide (ECPS) was produced by the mannase enzymatic hydrolysis process. The purpose of this research was to investigate the antimelanogenic activity of ECPS and CPS in B16F10 melanoma cells. The *in vitro* antioxidant activity was assessed by their ferric iron reducing power and DPPH free radical scavenging activities. The molecular mass distribution of polysaccharides was determined using SEC-MALLS-RI. CPS was successfully enzymatically degraded using mannase and the weighted average molecular weights of CPS and ECPS were 434.6 kDa and 211.7 kDa. The results of biological activity assays suggested that the enzymatically hydrolyzed polysaccharide had superior antimelanogenic activity and antioxidant effect than the original polysaccharide. ECPS exhibited antimelanogenic activity by down-regulating the expression of tyrosinase, MITF, and TRP-1 without cytotoxic effects in B16F10 melanoma cells. In conclusion, ECPS have the potential to become a skin whitening product.

## Introduction

Melanin is the L-tyrosine transformation end-product, which is the major determinant of hair and skin color and plays a vital role in protection against ultraviolet radiation injury (1). However, accumulation of melanin might be involved in abnormal pigmentation and result in hyperpigmentation of skin, melasma, solar melanosis, and ephelides (2). Biosynthesis of melanin involves a sequence of enzymatic and oxidative reactions and tyrosinase plays an important role in the process (3). The tyrosinase-related protein (TRP-1) facilitates the formation of DHICA oxidase in melanin biosynthetic pathway (4). Intracellular microphthalmia-associated transcription factor (MITF) is an important transcription regulator of melanin biosynthesis genes. MITF also participates in regulation of melanocyte pigmentation, proliferation, and differentiation (5). α-MSH-melanocortin 1 receptor signaling occurs in melanogenic specific enzymes, including TRP-1; tyrosinase is also regulated by the MITF (5). Many skin whitening agents exert the anti-melanogenic effects by regulation of tyrosinase expression or inhibitory effects on tyrosinase activity. Moreover, the intracellular antioxidant level and free radical production also have an effect on melanin content (6). Therefore, tyrosinase inhibitors and antioxidant compounds are often selected as skin whitening agents. *Cuscuta chinensis* Lam., called TuSiZi in Chinese, is a traditional Chinese medicine generally used as a functional food and known to enhance reproductive system ability (7). In recent years, some reports have indicated its use to treat freckles and vitiligo (8). Other reports have shown that it exerts a positive effect on skin protection (9), and induces the inhibition of tyrosinase activity (10).

Polysaccharides are the main constituents from the water extract of *C. chinensis* Lam. seed, which are considered to have anti-apoptosis (11) and immunological activities (12). Previous analytical results have indicated that *C. chinensis* Lam. polysaccharide is composed of fructose, mannose, xylose, and arabinose; mannose is the main sugar component (13). Many researchers have demonstrated that the viscosity (14), molecular weight (Mw) distributions (15), and monosaccharide proportion (16) of polysaccharides have a great effect on their bioactivity. Moreover, recent research has shown that degraded polysaccharides with low Mw exhibit higher antioxidant and tyrosinase-inhibiting activities than the original polysaccharide (17). Thus, the production of a low Mw polysaccharide from *C. chinensis* Lam. seed is necessary to improve its bioactivity. Among the different degradation processes, the major advantages of enzymatic degradation are the substrate specificity, high selectivity, and mild conditions, which produce hydrolysates with well-defined structures (18).

Based on these pharmacological studies, we speculated that *C. chinensis* polysaccharide (CPS) and enzymatically hydrolyzed *C. chinensis* polysaccharide (ECPS) might be effective botanical drugs for the improvement of hyperpigmentation. Mannase was used to obtain low Mw ECPS from seed. In addition, the antimelanogenesis and antioxidant activities of polysaccharides with different Mw were estimated, and the relationship between bioactivities and Mw of polysaccharides were investigated.

## Material and methods

### Reagents

Chemicals for enzyme and antioxidant activities were purchased from Sigma Co. (USA). All other reagents and chemicals were purchased from Aladdin (China).

### Preparation of CPS and ECPS

The medicinal materials of *Cuscuta chinensis* Lam seeds were provided from Guang Dong Feng Chun Pharmaceutical CO., LTD (China). About 500 g dry materials were powdered, and soaked with 1200 mL 80% ethanol for 24 h under room temperature to remove lipids, oligosaccharides, and colored materials. The pretreated samples were filtrated with cloth, and then the dried residue was extracted with 3000 mL water at 90°C for three times. The aqueous extracts were separated from the residue by centrifugation (4000 *g* for 5 min at 22°C) and then concentrated at 70°C under vacuum; the condensate was precipitated with 60% ethanol at 3°C for 24 h. Finally, the precipitate was deproteinated by the Sevag method, dialyzed with 3500 Da membrane, lyophilized, and then labeled *C. chinensis* polysaccharide (CPS).

The enzymatically hydrolyzed *C. chinensis* polysaccharide (ECPS) was obtained by hydrolysis with mannase (0.1% in sodium acetate buffer) in a mannase to substrate ratio of 5:1 (v/w) at 60°C, pH 4.5 for 6 h. Thereafter, the catalysis reaction was terminated in boiling water for 10 min. The reaction solution was centrifuged at 10,000 *g* for 15 min (4°C), the supernatant was collected for dialysis at 3°C for 3 days with a 3500 Da membrane to remove the small molecular substances, and was lyophilized.

The carbohydrate content was tested by the phenol-sulfuric acid method with glucose as the standard substance of a calibration curve.

### SEC-MALLS-RI measurement

Size exclusion chromatography (Waters, USA) combined with multi-angle laser light scattering detector (Wyatt, USA) and a refractive index detector (Waters, USA) (SEC-MALLS-RI) were used to detect weighted average molecular weights of polysaccharide. SEC-MALLS-RI was carried out with Phenomenex Polysep-GFC-Linear column (8 mm×300 mm); samples (2 mg/mL) were dissolved with mobile phase, which consisted of 0.1 M sodium chloride. The injection volume was 100 μL and flow was set at 0.7 mL/min.

### Mushroom tyrosinase inhibition assay

Mushroom tyrosinase inhibition (19) was performed as previously reported with modifications. Briefly, 25 μL of Kojic acid (positive control) or sample solutions (25 μL of 10 mM L-tyrosine, 25 μL of 0.5 mM L-DOPA, and 875 μL of 50 mM phosphate buffer (pH 6.5) solution) were mixed. Then, 38 μL of 2100 U/mL mushroom tyrosinase was added and vortexed. After 0.5-h incubation at 37°C, the absorbance was measured with a microplate reader at 475 nm (Thermo Fisher, USA). The inhibition percent of tyrosinase activity was calculated by the following formula: % tyrosinase inhibition = [(A-_control_ – A-_sample_) / A-_control_] × 100, where A-_control_ represents the absorbance at 475 nm without sample and A-_sample_ represents the absorbance at 475 nm with sample.

### Cell culture and viability assay

Murine B16F10 melanoma cells were purchased from Biochemistry and Cell Biology (China). Cells were maintained in Dulbecco's Modified Eagle Medium (DMEM) supplemented with 10% fetal bovine serum (FBS), 100 μg/mL streptomycin, and 100 IU/mL penicillin at 37°C in a humidified circumstance containing 5% CO_2_. Cells were seeded on culture plates and supplemented with different concentrations of samples and α-melanocyte stimulating hormone (α-MSH) for 72 h to measure the intracellular tyrosinase activity and quantitate melanin contents.

The 3-(4,5-dimethylthiazol-2-yl)-2,5-diphenyltetrazolium bromide (MTT) assay was carried out to test cell viability (20). Briefly, 96-well plates were seeded with murine B16F10 melanoma cells. A volume 50 μL of 2 mg/ml MTT was transferred into each well after treatment with 100 μL of different sample concentrations for 24 h. After 4-h incubation, the reaction was terminated and the dimethyl sulfoxide was added to dissolve the insoluble resultant. Absorbance was measured at 590 nm with the microplate reader.

### Measurement of melanin content

The detection of melanin content was carried out with the slightly modified method (21). After washing with iced PBS, melanoma cells (2 × 10^4^ cells per well) were seeded in a 96-well plate and incubated at 37°C for 48 h. Then, 100 μL NaOH (1N) was added to each well to dissolve melanoma cells at 80°C for 30 min. The lysate was centrifuged at 15,000 *g* for 15 min (4°C). Then, absorbance was measured with the microplate reader at 405 nm. All experiments were carried out in triplicate.

### Intracellular tyrosinase activity assay

Intracellular tyrosinase activity assay was carried out according to previous literature with minor modification (22). Briefly, melanoma cells were lysed with lysis buffer (1 mM PMSF, 1% Triton X-100, 20 mM sodium phosphate) by freeze-thawing. After centrifugation of the lysate at 15,000 *g* for 10 min (4°C), the protein content of the supernatant was determined by a bicinchoninic acid (BCA) assay. The supernatant protein (10 μg) was transferred into 100 μL of the reaction mixture (0.1% L-DOPA and 0.1 M phosphate buffer). After 60 min incubation at 37°C, tyrosinase activity was measured with the microplate reader at 450 nm. All the experiments were carried out in triplicate.

### Ferric iron reducing power

The ferric iron reducing power assay was performed according to previously published method with minor modifications (23). The different concentrations of samples (2 mL) or Vc (a positive control) were mixed with 2 mL potassium ferricyanide (1%, W/V) and 2 mL phosphate buffer (0.2 M, pH 6.8). After incubation at 50°C for 30 min, 2 mL trichloroacetic acid (10%, W/V) was transferred into the reaction mixture and centrifuged at 4000 *g* for 15 min (22°C). The supernatant (2 mL) was mixed with the mixture containing 2 mL distilled water and 0.4 mL FeCl_3_ (0.1%, W/V). After 10 min incubation at 37°C, the absorbance was measured with the microplate reader at 700 nm.

### DPPH radical-scavenging activity assay

The DPPH-scavenging activity assay was carried out as previously reported with some modifications (24). Briefly, 2 mL of the sample were added to 2 mL 0.1 mM DPPH solution and vortexed. After 30 min incubation in the dark, the absorbance was measured with the microplate reader at 517 nm.

### Protein expression analysis by western blot

After treatment with different concentrations of ECPS for 72 h, the cells were washed with PBS and lysed in RIPA buffer (150 mM NaCl in 50 mM pH 8.0 Tris-HCl, 0.5% sodium deoxycholate, 1.0% nonidet P-40, and 0.1% sodium dodecyl sulfate). After centrifugation at 10,000 *g* for 25 min (4°C), the supernatant of lysates was collected. The proteins were subjected to 12% SDS-PAGE and then transferred to polyvinylidene difluoride membrane. Blocking was carried out in Tris-buffered saline with Tween-20 and 2% skim milk powder (TBST), and then incubated for 12 h at 4°C. The primary antibodies used were: anti-β-actin (1:5000), anti-TRP-1 (1:500), anti-tyrosinase (1:500), and anti-MITF (1:1000). The primary antibodies were removed and the membranes were cleaned twice with TBST. After that, membranes with horseradish peroxidase-conjugated secondary antibody (Santa Cruz, USA) were incubated for 60 min at room temperature. The protein bands were washed with TBST again and visualized with ECL kit (Amersham Pharmacia Biotech, USA) using the UVP imaging system (UVP, USA).

### Statistical analysis

All results are reported as means±SD and the experiments were replicated three times. Comparisons between groups were estimated using ANOVA followed by Dunnett's test. Single comparisons between two groups were made by Student's *t*-test. All statistical analyses were made using SPSS software (version 16.0). P<0.05 was usually considered to be statistically significant.

## Results

### Mw and total polysaccharides of ECPS and CPS

**Figure 1.**
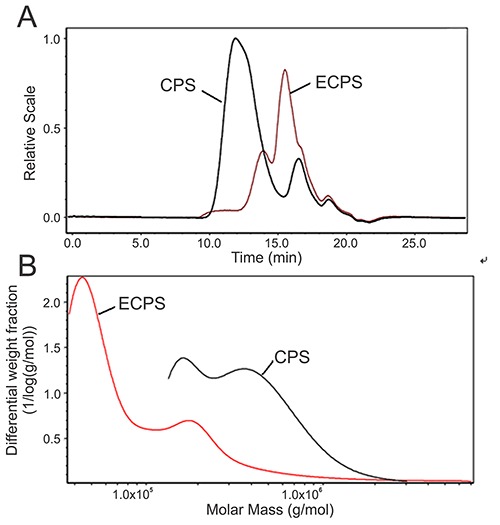
SEC-MALLS-relative intensity result for enzymatically hydrolyzed *Cuscuta chinensis* polysaccharides (ECPS) and *Cuscuta chinensis* polysaccharides (CPS) dissolved in 0.1 M sodium chloride solution at 24°C. *A*, Relative intensity. *B*, Molar mass distribution.

The total polysaccharide contents of ECPS and CPS measured by phenol-sulfuric acid assay were 89.17 and 90.26%, respectively. Meanwhile, the Mw of ECPS and CPS were measured by SEC-MALLS-RI. The Mw of ECPS was 211.7 kDa, which was lower than CPS (434.6 kDa). Figure 1A shows the relative intensity (RI) for ECPS and CPS; after enzymatic hydrolysis by mannase, the peak retention time of ECPS was longer than that of CPS. As displayed in Figure 1B, the differential weight fractions of polysaccharides were portrayed as the function of molar mass for samples. The molar mass distribution of polysaccharide changed significantly by enzymatic hydrolysis. The differential weight fraction of ECPS in the low Mw region increased, which suggested that the CPS was enzymatically degraded into low Mw polysaccharide.

### Antioxidant activities of polysaccharides

**Figure 2.**
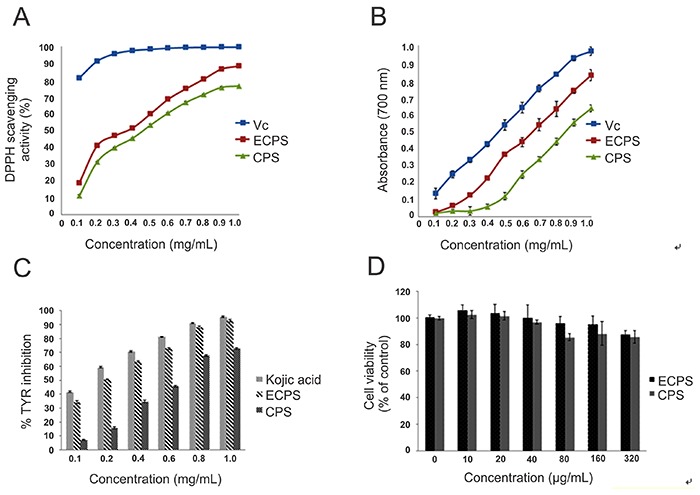
Antioxidant and anti-tyrosinase effects of polysaccharide fragments. *A,* DDPH scavenging activity. *B*, Ferric iron reducing power. *C*, Tyrosinase (TYR) inhibition rates compared with positive control (Kojic acid). *D*, Effects of ECPS and CPS on B16F10 cells viability. Data are reported as means±SD (n=3). ECPS: enzymatically hydrolyzed *Cuscuta chinensis* polysaccharides; CPD: *Cuscuta chinensis* polysaccharides; Vc: positive control.

The DPPH free-radical scavenging abilities of ECPS and CPS are reported in Figure 2A. The free-radical scavenging activities of polysaccharide samples and Vc exhibited a dose-dependent activity. In the current study, the free-radical scavenging ability of CPS was lower than that of ECPS. However, both exhibited lower free-radical scavenging effect than the positive sample. The IC_50_ values of ECPS and CPS were 0.39 and 0.51 mg/mL, respectively. As displayed in Figure 2B, the total antioxidant activity can be assessed by testing the ferric iron reducing power. The concentrations varied from 0.1 to 1 mg/mL; both polysaccharide samples and Vc presented antioxidant activity in a dose-dependent manner. Moreover, the absorbance value of ECPS was always higher than that of CPS at the same concentration.

### Effect of ECPS and CPS on mushroom tyrosinase activity and cell viability

As shown in Figure 2C, the tyrosinase inhibitory activity of polysaccharides (0.1∼1 mg/mL) presented a dose-dependent relationship. Moreover, the inhibitory effect of ECPS was always higher than that of CPS at the same concentration. The MTT assay was performed to assess the cytotoxic effects of ECPS and CPS in B16F10 melanoma cells. As displayed in Figure 2D, there were no significant changes in B16F10 cell viability with different concentrations (0∼320 μg/mL) of ECPS and CPS. Based on these results, we used these concentration ranges in further research.

### Effect of ECPS and CPS on intracellular tyrosinase activity and melanin contents

**Figure 3.**
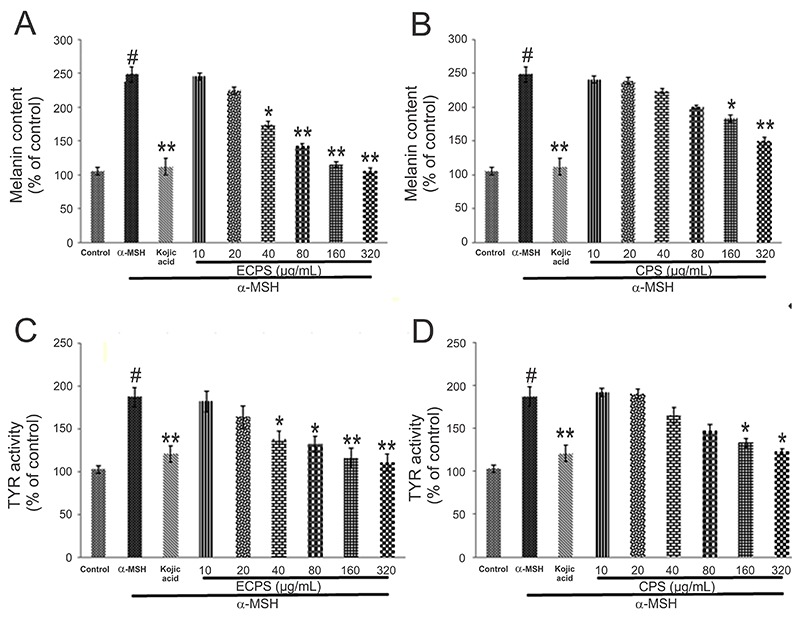
Effects of enzymatically hydrolyzed *Cuscuta chinensis* polysaccharides (ECPS) and *Cuscuta chinensis* polysaccharides (CPS) on B16F10 cells. Melanin content and tyrosinase (TYR) activity of melanoma cells were measured after ECPS (*A* and *C*) and CPS (*B* and *D*) treatment. Kojic acid (160 μg/mL) was used as positive control. Data are reported as means±SD. ^#^P<0.01 compared to control group; *P<0.05, **P<0.01 compared to α-melanocyte stimulating hormone (α-MSH) group (ANOVA followed by Dunnett's test).

To compare the effects of ECPS and CPS on the activity of intracellular tyrosinase and melanogenesis in B16F10 melanoma cell model, the inhibitory potency of ECPS and CPS on melanin content and tyrosinase activity in α-MSH-stimulated B16F10 cells were examined. As shown in Figure 3, melanin content and tyrosinase activity of B16F10 cells were significantly increased when compared to the unstimulated B16F10 cells (P< 0.01). At concentrations of 40 μg/mL (ECPS) and 160 μg/mL (CPS), the increase of melanin contents could be mitigated in a dose-dependent manner (P<0.01 and P<0.05). Similarly, treatment with ECPS (40 μg/mL) and CPS (160 μg/mL) suppressed the tyrosinase activity of B16F10 cells (P<0.01 and P<0.05). Moreover, ECPS exhibited higher tyrosinase inhibitory activity on melanogenesis than CPS. ECPS (160 and 320 μg/mL) exerted antimelanogenesis effect comparable to the positive control (Kojic acid), which is widely used as skin whitening bioactive compound.

### Effect of ECPS on tyrosinase, MITF, and TRP-1 protein levels in B16F10 cells

**Figure 4.**
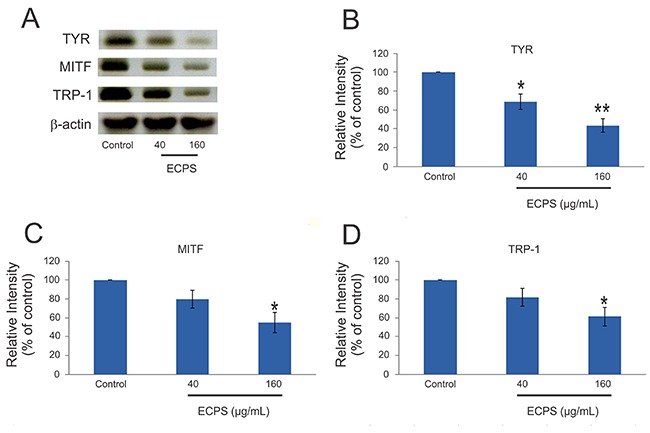
Protein levels of tyrosinase (TYR), microphthalmia-associated transcription factor (MITF) and tyrosinase-related protein (TRP-1) were examined by western blots (*A*). The ratios of TYP/β-actin, MITF/β-actin, and TRP-1/β-actin are reported in panels *B*-*D* as means±SD. *P<0.05, **P<0.01 compared to control group (ANOVA followed by Dunnett's test). ECPS: enzymatically hydrolyzed *Cuscuta chinensis* polysaccharides.

As shown in Figure 4, the ECPS significantly decreased tyrosinase, MITF, and TRP-1 protein expression levels in B16F10 cells in a dose-dependent manner (P<0.05 and P<0.01). These results show that ECPS inhibited the expression of tyrosinase by down-regulating protein expression of TRP-1 and MITF.

## Discussion

The natural polysaccharides from *C. chinensis* have received attention attributed to the good effects on tyrosinase inhibition, free radical scavenging, and skin protection (25–27). However, little research has focused on the antimelanogenesis activity of enzymatic modification of polysaccharides. Previous research has demonstrated that degraded polysaccharides by enzymatic hydrolysis process exhibited superior free radical scavenging effect (28). Moreover, the biological activities of polysaccharides are closely related to their Mw distributions. Theoretically, low Mw polysaccharides are more active than high Mw polysaccharides due to their high penetration property on cell membranes (29,30). However, the antimelanogenesis effect of ECPS on B16F10 cells had not yet been studied. The low Mw polysaccharide was prepared by enzymatic hydrolysis with mannase.

Oxidative stress can produce excessive free radicals and lead to oxidative injury. Previous studies have proven that skin disease is closely related to accumulation of free radicals (31). Moreover, excessive free radicals play a vital role in suppressing melanogenesis of melanoma cells and growth of melanocytes (32). Tyrosinase is a multifunctional oxidant enzyme that contains bronze and is vital in promoting melanin biosynthesis (33). However, skin pigmentation and various skin diseases are closely related to the accumulation of melanin and cause a serious esthetic problem (34).

Active ingredients with antioxidant and anti-tyrosinase abilities can exert skin protection and inhibit melanogenesis (35). Our results have demonstrated that the lower Mw of enzymatically modified polysaccharides exhibited superior antioxidant and anti-tyrosinase activities than original polysaccharides *in vitro*. The improvement is attributed to the greater surface area and better water solubility, which was consistent with a previous study (17) that showed that the degraded polysaccharide from *Sargassum fusiforme* possesses superior anti-tyrosinase activity and antioxidant activity than the original polysaccharide.

Normal melanocytes lie at the junction of the epidermis and dermis of the skin and generate melanin, which is transferred to keratinocytes (36). In the present study, the murine B16F10 melanoma cells were used because they possess melanogenic mechanism, are known to have intracellular tyrosinase, and can generate melanin, which are related to α-MSH stimulation and melanogenesis (37). Tyrosinase activity, melanin content, and cell viability were the *in vitro* assays used to screen antimelanogenesis in present study. CPS and ECPS exhibited a dose-dependent inhibitory effect on tyrosinase activity and melanin synthesis in B16F10 cells. ECPS showed a stronger anti-melanin synthesis and anti-tyrosinase effect.

Tyrosinase-related protein-1 (TRP-1) and tyrosinase play a vital role in melanin biosynthesis and melanogenesis pathways (38). MITF is a cellular transcription factor of the tyrosinase gene, which takes part in melanogenesis. Usually, the activation of TRP-1 and tyrosinase enhances MITF protein expression and causes the increase of melanin synthesis (39). Thus, skin whitening agents may have the property of inhibiting the signaling pathway involved in the activation of TYP-1 or tyrosinase. Therefore, we investigated the effects of ECPS on TRP-1, cellular tyrosinase and MITF protein expressions to study the mechanisms underlying the inhibition of tyrosinase activity and melanogenesis. The results of western bolt assay showed that ECPS suppressed the expression of TRP-1, tyrosinase, and MITF in B16F10 cells and implied that ECPS decreased melanogenesis by down-regulating tyrosinase, MITF, and TRP-1 expression in B16F10 melanoma cells. The result was in accordance with a previous study showing that the aqueous extract from *Cuscuta japonica* seed significantly inhibited α-MSH-induced melanin synthesis and tyrosinase activity by suppressing p38 MAPK phosphorylation, inhibiting cAMP levels, and subsequently decreasing the expression of TRP and MITF (40).

In summary, the enzymatically modified polysaccharide possessed superior antioxidant and antimelanogenic effects than the original polysaccharide. Furthermore, this antimelanogenic effect of ECPS was mediated by the suppression of TRP-1, tyrosinase, and MITF expression in murine B16F10 cells. ECPS can be applicable for use in the fields of cosmetic and medicine products.
